# Preventing Mental Ill-Health: Informing Public Health Planning and Mental Health Practice

**DOI:** 10.1192/pb.bp.114.046920

**Published:** 2015-02

**Authors:** Benjamin Bouquet

**Figure:**
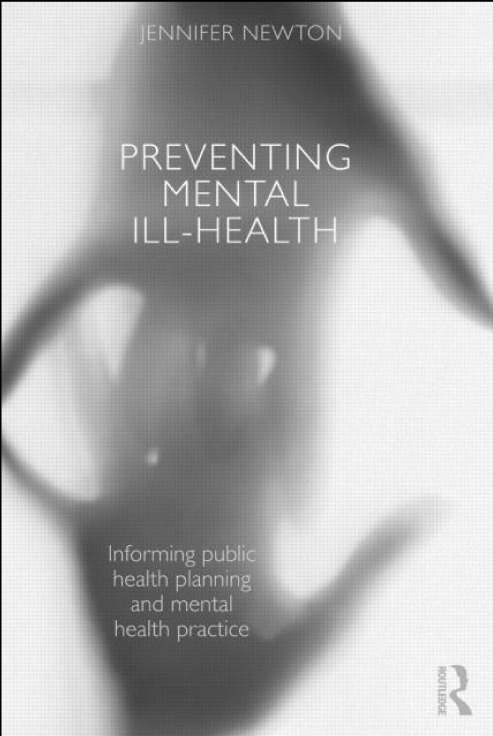


This book covers an ambitious breadth of material concerning the definition, determinants and interventions for prevention of mental ill health. The sheer scale of material covered means that the reader should not expect an in-depth critique of all the evidence presented and this can pose questions around the methodology and conclusions of studies. The author notes that she is an agnostic entering the houses of such new religions as biological psychiatry and positive psychology. Their differing perspectives occasionally lead to a conflict in argument, which is not always resolved. Psychiatric labels are defended for their contribution to research, while later it is reported that improved understanding of schizophrenia has derived from breaking the diagnosis down into constituent symptoms.

My favourite statistic from the book is the reported finding that 2.4% of women from a Basque-speaking rural area screened positive for depression compared with 11% of women in a Spanish-speaking village in the Basque region. The degree of integration in each community is cited as an explanation, echoing the famous theories of Emile Durkheim around suicides and social cohesion.

Despite a thought-provoking chapter on ‘society, status and participation’, the focus of the book is very much on the individual’s place within society. There is a good discussion of negative consequences of housing policy and a look at unemployment and inequality, but in terms of social determinants of mental health, I was left wondering how to build societies with the kind of integration that seems so protective. For the individual, the take-home message is that what matters is to feel loved, safe, valued and in control.

